# Institutional Performance and Vote Buying in India

**DOI:** 10.1007/s12116-017-9254-x

**Published:** 2017-11-07

**Authors:** Oliver Heath, Louise Tillin

**Affiliations:** 1 Department of Politics and IR, Royal Holloway, University of London, Surrey, TW20 0EX UK; 2 King’s India Institute, King’s College London, London, WC2R 2LS UK

**Keywords:** Vote buying, Clientelism, Institutional performance, India

## Abstract

Inefficient and corrupt institutions provide an incentive for citizens to focus on short causal chains, which prize instant benefits from direct, clientelist exchanges over the promise of uncertain and distant programmatic rewards. Drawing on a tightly controlled comparison arising from the bifurcation of a state within the Indian federal system into two units that have demonstrated marked differences in institutional development post division, and a survey administered across the new state boundary, we show that citizens are more responsive to small inducements in weak institutional settings where the delivery of basic goods by the state is less certain, but that these institutional effects weaken as the size of the inducement increases.

## Introduction

When do voters ‘sell their vote’ and how is their decision to do so influenced by the institutional context? In this article, we examine how improved institutional performance in public service delivery affects citizen responsiveness to clientelist appeals. We argue that where institutions are inefficient and function badly, citizens have greater incentive to prize the short-term benefits that clientelist exchanges provide than the long-term—yet uncertain—goods that are promised by programmatic policies. Poor institutional performance therefore makes the prospect of a bag of goodies in the hand today more attractive than the promise of distributive public policy tomorrow. But in situations where institutional reforms make the delivery of basic goods by the state more reliable, citizens may become less responsive to vote buying.

In developing our hypotheses, we build on two key insights from the literature. First, citizens respond to clientelist appeals because they are risk averse, preferring direct, instant clientelist benefits over indirect, programmatic policies promising uncertain and distant rewards to voters (see, for example Desposato 2007; Kitschelt 2000; Kitschelt and Kselman 2013; Scott 1977; Wantchekon 2003). Second, clientelist appeals have diminishing marginal utility: thus, poor people value a handout more highly than do wealthy people; hence, if one is going to hand out material inducements, one will target the poor (Dixit and Londregan 1996; Calvo and Murillo 2004; Stokes et al. 2013).

Previous research has tended to examine both these factors from the perspective of poverty and education. But—as we show—the institutional context can also have a strong bearing on the nature of this calculus. If institutions do not function well, and are leaky, then the probability of ever receiving the promised benefit of a programmatic policy is extremely low. In this situation, rational voters will discount the future and the prospect of short-term clientelist goods will be more attractive. We would therefore expect citizens to be more responsive to vote buying in such a setting. However, when institutions function better, voters can see the link between policy promises and policy implementation and so will be less likely to sacrifice their preferred policy outcome for a short-term pay-off.

In addition, the diminishing marginal utility of clientelist appeals has tended to be regarded as a function of citizen income: as people become wealthier, they will value less the fixed price of a good that they are offered. Or put another way, the greater the value of the good voters are offered, the less difference there will be between whether rich people and poor people are responsive to the inducement. We show that institutional context can also influence the marginal utility of vote buying. When public services function badly, people will sell their vote for relatively little, but as institutions perform better the cost of vote buying also increases. This implies that the greater the value of the good voters are offered, the less difference there will be between whether people are prepared to sell their vote in well-performing institutional settings and badly performing institutional settings. Therefore, we expect to see larger institutional effects on small inducements than large inducements.

In order to test these propositions, we take advantage of a tightly controlled comparison in central India made possible by the division of the state (federal sub-unit) of Madhya Pradesh in 2000, leading to the formation of the new state of Chhattisgarh. Given that a natural experiment is an extremely difficult (if not impossible) research design to execute for our research question, this approach is arguably the next best alternative. By carrying out a cross-border survey and studying villages on either side of a newly created state border, we are able to exert a high degree of control over factors commonly associated with clientelism, such as poverty, political competition, socioeconomic structure, ethnic and kinship relations, and administrative history. Since bifurcation, however, political leaders in the two states have made different decisions about how to work with the local bureaucracy to shape the performance of institutions that are critical for the delivery of public services. Their different strategies have led to substantial variation in the delivery of social welfare programmes, particularly those concerned with basic food provision and employment. Inhabitants living on either side of the border are thus exposed to different institutional contexts. Those living on the Chhattisgarh side of the border have experienced relatively more efficient, universal, and easy-to-access social welfare programmes compared to people living on the Madhya Pradesh side of the border where important social programmes remain (inefficiently) targeted, leaky, and subject to local political intermediation. Villages and inhabitants on the immediate side of either border are similar in practically every other way apart from these institutional settings.

In taking advantage of this tightly controlled comparison, we join a number of other researchers (see, for example Laitin 1986; Miguel 2004; Miles and Rochefort 1991; Posner 2004) who have also exploited the partitioning of ethnic groups by administrative boundaries to study how similar social groups respond to different social and political environments. We can thus explore how groups with similar socioeconomic backgrounds respond to clientelist appeals in different institutional environments, while holding constant the most important factors associated with the public responsiveness to clientelism across the groups.

## Institutional Performance and Clientelism

India is often regarded as a ‘patronage democracy’ (Chandra 2004). Vast amounts of money continue to circulate during Indian elections. In the run-up to elections in the state of Bihar in 2015, journalists reported that ‘almost 17 crore [$2.5million] in cash’ and ‘1.5 lakh [150,000] litres of liquor’ had been seized under the electoral code of conduct in a state where ‘cash and liquor are commonly used…to influence voters’ (Pandey 2015). The *Wall Street Journal* asked during India’s 2014 General Elections, ‘The big question for some voters … isn’t who will win, it is how much candidates will dole out in cash, alcohol and other goodies to bag their support’ (Mandhana and Agarwal 2014). These reports reflect a widely held popular perception that vote buying plays an important role in determining voter behaviour.

In this article, we focus on this material dimension of clientelism, rather than the less tangible—although still important—longer-term relationships that are also embedded within vote buying exchanges. Following Schaffer (2007, 5), we define ‘vote buying’ as the offer of particularistic material goods (such as cash, food, clothes, household items) to individuals or households at election time in an attempt to influence election outcomes. Despite the prevalence of clientelist exchanges of this type in many democracies around the world, we still know comparatively little about why parties pursue this type of strategy and why voters respond to them. The literature identifies several possible determinants of clientelism. These include economic development and public responsiveness to clientelist appeals (Wantchekon 2003; Brusco et al. 2004; Weitz-Shapiro 2012), state institutions and politicians’ access to public resources (Hicken 2011), and political competition and politicians’ incentives to make clientelist appeals (Kitschelt and Wilkinson 2007). Other explanations include the role of cultural norms such as reciprocity (Auyero 2000; Putnam 1993), ethnicity (Chandra 2004; Kitschelt and Wilkinson 2007), and political institutions such as regime type, campaign finance regulations, electoral systems, and ballot design (Golden 2003; van de Walle 2003; Roniger 2004; Lehoucq and Molina 2002; Brusco et al. 2004; Hicken 2007).

Although scholars have proposed a wide variety of different explanations for the prevalence of clientelism, the causal mechanisms at work have been contested and empirical evidence has been mixed. While some controversies have been resolved, others remain. For example, whereas there is now widespread agreement in the literature that economic (under-)development is an important reason for clientelism, the relationship between institutional performance and clientelism is still a source of lively debate. Although it is clear that institutional legacies are important, it is far less clear how they are important and in what ways.

The performance and autonomy of bureaucracies may influence elite incentives to pursue clientelist exchanges. Shefter (1977, 1993) argues that in administrative systems where a high degree of bureaucratic autonomy precedes democratisation, the ability of politicians to divert state resources towards clientelist strategies is greatly reduced. Furthermore, he suggests that if political parties are incumbents and therefore have access to state resources, they are more likely to rely on clientelism than ‘outsider’ or challenger parties who are not in positions of power in the existing regime and are therefore forced to rely on programmatic or ideological appeals to fight their way to power (see also van de Walle (2003) on the importance for parties of winning ‘founding’ elections). Along similar lines, Huber and McCarty (2004) argue that where bureaucratic capacity is lower, politicians will find it harder to achieve their policy goals and will have greater incentives to politicise the bureaucracy, increasing the prevalence of clientelism.

Keefer (2006) posits the alternative view that once parties win power, there may be little to prevent them politicising the bureaucracy to turn it into a source of patronage. Political interference with once autonomous bureaucracies is not uncommon (Hicken 2001; Baxter et al. 2002; Kitschelt and Wilkinson 2007). Keefer and Vlaicu (2008) thus argue that whether or not politicians pursue clientelist strategies depends instead upon their strategies for building political credibility rather than the prior quality of institutions. They argue that it is costly for politicians to build a reputation for political credibility via public policy commitments: ‘Politicians must expend resources to reach voters with their promises, to allow voters to monitor the fulfilment of promises, and to ensure that voters turn out on election day’ (ibid 372). Politicians can opt out of these expenditures by relying on intermediaries (or brokers) who already have a ‘customary trust relationship’ with some groups of voters.

Empirical studies have shown that there is substantial variation in the extent to which political leaders across India rely primarily on clientelist strategies to mobilise votes. Many leaders recognise the need to supplement traditional clientelist strategies with programmatic activities or ‘post-clientelist’ strategies that are not implemented in a particularistic or discretionary manner (Wilkinson 2007; Manor 2010; Wyatt 2013). With a growing private sector and larger middle class, the electorate may be less dependent on the state, while the growth of the mass media has also increased scrutiny of corruption (Wilkinson 2007).

In this article, we build on these insights by examining the issues from a slightly different perspective. Rather than examining how the performance of bureaucracies influences elite incentives to pursue clientelist exchanges, or how elites politicise the bureaucracy to turn it into a source of patronage, we examine how the performance of bureaucratic institutions affects citizens’ responsiveness to clientelist appeals and how elites can reform the bureaucracy to make it more effective and less corrupt. We do so with reference to the delivery of public services, and the delivery of subsidised food via the Public Distribution System specifically. By institutional performance, we are primarily concerned with the record of the bureaucracy in performing routine tasks of administration that affect the delivery of government policies and programmes. By institutions, we mean organisations within the state and federal bureaucracy and the norms and practices shared among officials.^1^

## Research Design

Clientelist practices may emerge in contexts of weak institutional capacity and may also undermine institutional capacity. That is, clientelism may be both a cause and a consequence of institutional performance. Indeed, a large body of research shows that clientelism is at best inefficient and at worst corrupt, with clientelist systems exhibiting lower primary school enrolment rates, less effective use of public resources, and more corrupt business practices than programmatic systems (Keefer 2006, 2007; Hicken and Simmons 2008; Singer 2009). Moreover, both the prevalence of clientelism and the performance of institutions may be co-determined by historical legacies relating to the development of the bureaucracy. This means that it is very difficult to examine the relationship between institutional capacity and clientelism, and attempts to do so (e.g. Bustikova and Corduneanu-Huci 2009) have been criticised for failing to take into account reciprocal causation (see Hicken 2011).

We address this problem by carrying out a carefully constructed comparison which exploits the division of Madhya Pradesh into two states. Madhya Pradesh and Chhattisgarh share many economic, developmental, political, and cultural similarities and until 2000 were part of the same state. They are predominantly rural and Hindi speaking, with large populations of ‘Scheduled Tribes’ or indigenous communities. They have a common political history of Congress Party dominance, challenged in recent decades by the ascendancy of the Hindu nationalist BJP which has consistently won all state legislative assembly elections in both states since 2003. Both have two-party systems and unlike other Indian states have not seen the emergence of strong regional parties. Both states are characterised by high levels of poverty and under-nutrition. Five years after their bifurcation, in both states almost 50% of the population was classified as living below the poverty line compared to 37% at the all-India level (Planning Commission 2012). Our empirical strategy takes advantage of the fact that villages on the Madhya Pradesh side of the border are very similar to villages on the Chhattisgarh side of the border—save for the administrative zone under which they fall.

India’s constitution gives the federal government the ability to divide or change the boundaries of states within the federation on the basis of a simple parliamentary majority. Unlike most other instances in which new states have been created in India, the bifurcation of Madhya Pradesh did not respond to strong popular mobilisation demanding statehood. The division of Madhya Pradesh arose from inter-elite contestation, rather than pressure from below (Tillin 2013). This is in contrast to the other two states which were created elsewhere in India at the same time, Jharkhand and Uttarakhand, where there had been long-run social mobilisation for greater regional autonomy in the form of statehood and where patterns of political competition have diverged from their parent states since their bifurcation.

**Fig. 1:**
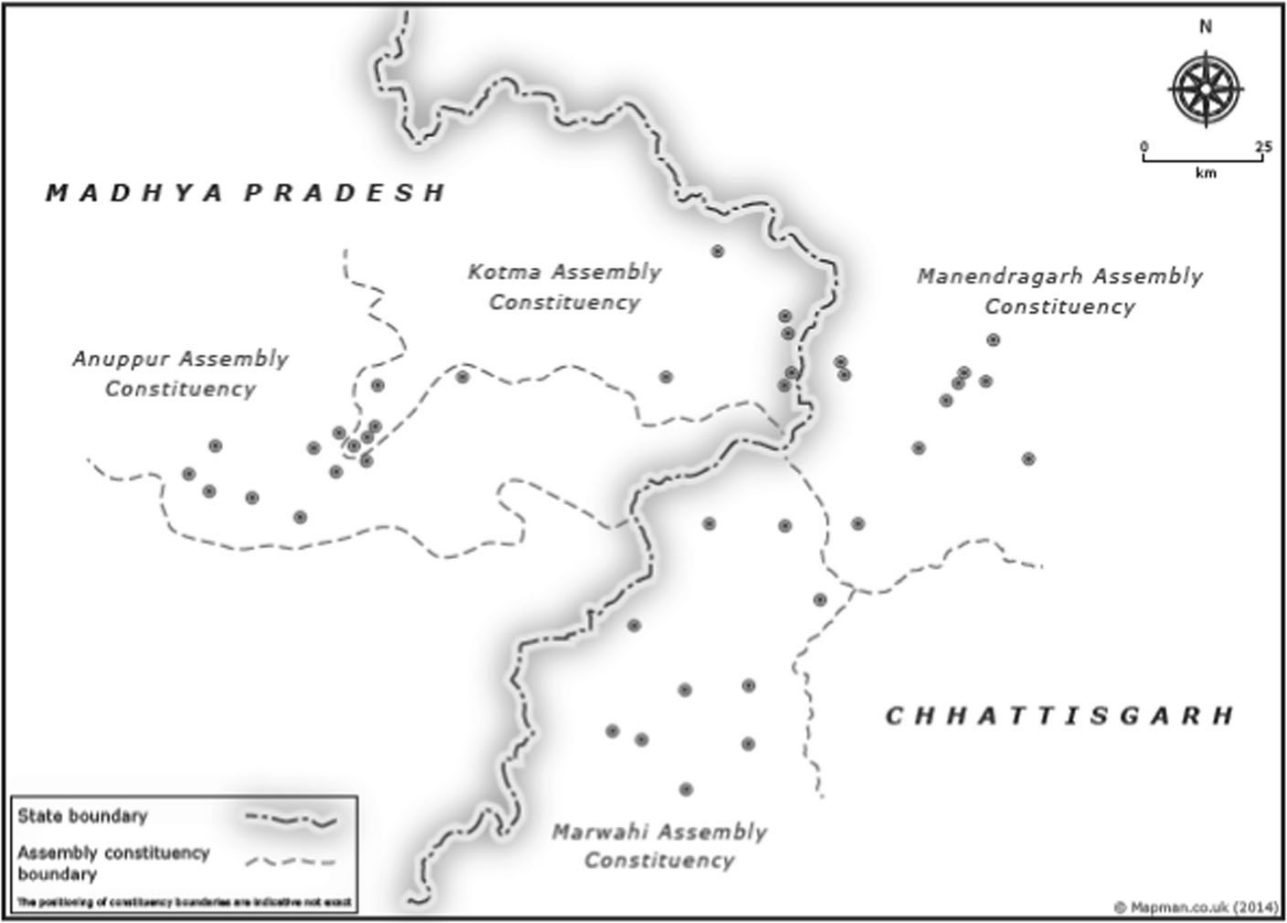
Research site

While the creation of the new state of Chhattisgarh was more top-down than other instances of state creation, the redrawing of state boundaries was not entirely random since the new state borders follow the line of earlier district boundaries. In the area covered by Marwahi assembly constituency (see Fig. 1), the state border followed the contour of the district boundary in the old colonial-era Central Provinces and Berar province, and to the north the border followed district boundaries that encompassed areas governed by earlier princely states. The process of bifurcation was striking in that there was little contestation about which districts should be included in Chhattisgarh. The region of Madhya Pradesh that became present-day Chhattisgarh was regarded as a backwater of the parent state. It was poor and under-developed, despite the presence of natural resources. Unlike the cases of Jharkhand and Uttarakhand (and the more recent case of Telangana), there was no backlash from the parent state against the proposed bifurcation and little sustained dispute as to where the new state border should fall. In fact, the first resolution in favour of creating the state was passed without any major opposition by the Madhya Pradesh state assembly in 1994 (Tillin 2013, 153).

The districts sampled in our study, which all have substantial tribal populations, were mostly indirectly ruled by various princely states during the colonial era, and thus bear many similarities to each other in terms of their longer-term histories of administration. Moreover, the borders were not drawn to enhance the political advantage of incumbent elites on either side, and the districts on either side of the border remain nearly identical in terms of party competition and electoral outcomes. Thus, the division of the two states was—unlike many other instances of state creation in India—a largely top-down administrative reform that did not reflect popular mobilisation or different patterns of electoral politics on the ground. Populations living immediately adjacent to the state border were divided by a new administrative boundary that they had not played a role in demanding or constructing.

As Table 1 shows, at the point of bifurcation, the villages on either side of the state border were very similar to each other in social and political terms, and in institutional terms with respect to the provision of public services. While the proportion of Scheduled Tribes was higher in the Shahdol district of Madhya Pradesh in 2001 than in the Chhattisgarh districts, the proportion of Scheduled Castes was higher on the Chhattisgarh side.^2^ Over 40% of the population in the districts on either side of the border come from these socioeconomically disadvantaged groups. Politically, the assembly segments on either side of the border were both characterised by competitive contests between the BJP and Congress, with similar levels of voter turnout suggesting little difference in civic participation. And institutionally, the proportion of villages with access to education and health services, water and electricity, and transportation links was very similar on either side of the border, reflecting the common administrative history the two new states shared.

**Table 1:** Pre-bifurcation balance checks

	Chhattisgarh	Madhya Pradesh
Demographics		
Literacy	52	49
Sex ratio^a^	959	956
In work	44	43
Scheduled tribes	25	44
Scheduled castes	16	7
Service delivery		
Safe drinking water	98	100
Electricity for domestic purposes	56	52
Primary school	89	94
Secondary school	13	12
Primary health centre	15	10
Bus services	14	21
Paved approach road	34	33
Politics		
Turnout	54%	49%
BJP	43%	44%
Congress	41%	36%
JD	5%	1%
BSP	4%	6%

^a^Women per 1000 men. Demographic and Service Delivery data comes from district area profiles, Census of India 2001. The districts are Shahdol in Madhya Pradesh, and Bilaspur and Koriya in Chhattisgarh. Political data comes from 1998 Vidhan Sabha elections, Election Commission of India

However, despite their common administrative histories and shared developmental challenges, since bifurcation the political leadership of the two states has pursued markedly different approaches to public administration, particularly with regard to the delivery of welfare programmes. In Chhattisgarh, the state’s top political leadership made the strategic decision to improve the efficiency of social transfer programmes, in particular the Public Distribution System (PDS) through which subsidised food is distributed, and the Mahatma Gandhi National Rural Employment Guarantee Scheme (MGNREGS) through which rural households can demand up to 100 days of employment per year on public works. By contrast in Madhya Pradesh, no such strategic decisions were made by the political leadership. Food subsidies, in particular, continued to function as a major system of patronage dispersal.

Throughout India, the Public Distribution System has been known for high levels of corruption. A Planning Commission survey in 2004 estimated that 58% of foodgrains do not reach their intended beneficiaries (Saxena 2012). Common complaints about the operation of the PDS relate to the sale of foodgrains on the black market, the adulteration of the foodgrains sold through ‘fair price shops’, unaccountable local shopkeepers who do not open at regular hours and frequently claim not to have received supplies, and local bureaucratic corruption or inefficiency which frequently excludes the poorest from possession of the requisite ‘ration card’ (see summary in Pritchard et al. 2013, 110–1). For these reasons, the PDS has been the subject of vigorous national debate and reform efforts. India’s Supreme Court issued a series of legal orders from 2001 onwards seeking enforcement of the ‘right to food’. These orders placed a legal requirement on all states to improve the performance of the Public Distribution System. More recently, after a lengthy political debate, a new National Food Security Act was passed in late 2013 providing statutory backing to a ‘right to food’, and stipulating new requirements for food subsidies and their delivery. Yet, states have demonstrated substantial variation in how they have approached edicts to improve the efficiency of the Public Distribution System.^3^

Since 2003, the state government in Chhattisgarh has embarked on the most far-reaching reforms to the PDS of any state in India. At the heart of these reforms was an overhaul of the delivery of subsidised food through a combination of reforms to delivery mechanisms, computerisation, and an expansion of the beneficiary pool to transform a programme that had been targeted towards the ‘below poverty line’ (BPL) population into a quasi-universal programme. It brought ‘fair price shops’ and the transportation companies which move foodgrains between field, rice mill, warehouse, and shop, back into public ownership. These decisions triggered over 400 court cases, and required the state’s top political leadership to withstand pressure from private traders, an important constituency for the incumbent BJP. Licences to run fair price shops were then granted to local elected councils, self-help groups, and cooperative societies in an attempt to improve their accountability to local communities. Subsequently, the Chief Minister, Raman Singh, decided to substantially expand the proportion of the population with access to heavily subsidised food by launching a new ‘Chief Minister’s Food Security Scheme’ which granted 35 kg of rice at just 2 rupees per kilogramme to poor families, and at 1 rupee per kilogramme to the poorest. Simultaneously, senior civil servants were empowered to undertake efficiency reforms through computerisation and enhanced transparency mechanisms, including the use of GPS tracking of trucks moving foodgrains, sending SMS messages to local villagers to inform them of the date new foodgrains would be delivered to their local shop, and using a centralised computerised database to reissue ‘ration cards’.^4^

The reforms have had a dramatic impact on preventing leakages of foodgrains and ensuring foodgrains reach the final mile to the fair price shop, as well as ensuring that access to ration cards is not dependent on the discretion of local officials (see also Patnaik 2010; Puri 2012). By increasing transparency and reducing corruption, they have changed the way that these welfare programmes are delivered in Chhattisgarh and—as our survey results demonstrate—have had a pronounced impact on the delivery of, and satisfaction with, the operation of the Public Distribution System specifically. Chhattisgarh is also considered to be one of the more effective states at implementing the Mahatma Gandhi National Rural Employment Guarantee Scheme, the largest social protection programme introduced in India in the last decade (Dreze and Khera 2014).

By contrast, in Madhya Pradesh, access to the same schemes is much more unreliable and bureaucratic corruption continues to play a critical role in determining access. The Public Distribution System in particular is notoriously ‘leaky’, foodgrains are siphoned off at various stages of the system, and the poor have extremely unreliable access, compounded by the fact they frequently do not possess the requisite ration card to secure their entitlements in the first place. While the state government also attempted to initiate reforms to the Public Distribution System, the reform process was more confused and did not receive clear direction from the political leadership. Reforms in Madhya Pradesh focused more on the question of dealing with ‘inclusion’ errors—with people who should not have Below Poverty Line ration cards but do—rather than with ‘exclusion errors’. The thrust was largely technocratic with a private consortium hired, on the basis of a contract that was veiled in secrecy, to create a biometric ration card database which was intended to be linked to a system of food coupons in the future. At the time of our survey in late 2013, there had been very little attempt to improve transparency or accountability, and the leadership necessary to overcome local political and bureaucratic resistance to reform was not evident. Bureaucratic malpractice was widespread. Local villagers frequently had to pay bribes to get a ration card, foodgrains were often mixed with impurities, and the poorest were often denied the correct level of entitlement—as the survey evidence we present below demonstrates. This means that the PDS remained a vehicle for patronage, captured by vested interests, rather than for the effective delivery of foodgrains to the food insecure.

It is worth emphasising here that the differences between the two states are of degree and not absolute. Patron-client relations continue to exist in Chhattisgarh but have been supplemented by more successful instances of programmatic policy delivery. Equally, Madhya Pradesh is far from the most feckless of state governments (Jenkins and Manor 2015). But there are stark differences in outcomes in terms of the performance of key areas of social policy to do with food and employment. According to data collected as part of the ‘PEEP survey’ by Dreze and Khera (2014), access to the PDS scheme among eligible recipients is much higher in Chhattisgarh than it is in Madhya Pradesh (99 vs 49%) and the average number of days worked per household registered on the MGNREGS is much higher in Chhattisgarh than in Madhya Pradesh (34 vs 8).

Although it is beyond the scope of this article to fully explain why the two states have pursued such different strategies, one reason may be to do with electoral strategy and the ways in which the respective BJP leaders have sought to achieve what Keefer and Vlaicu (2008) term ‘political credibility’, particularly among the rural poor. The expansion, and improved operation, of the PDS has been central to the election campaigns run by Chief Minister Raman Singh in Chhattisgarh. By contrast, the Chief Minister of Madhya Pradesh Shivraj Singh Chauhan has focused more on the projection of a pro-farmer administration that builds on his rustic background and has seen Madhya Pradesh become one of the leading states for wheat procurement (Krishnamurthy 2012; Tillin et al. 2015). While any explanation for why the political leaders adopted such different strategies must be treated as somewhat speculative, the crucial point is that under these leaders the two states diverged in terms of their institutional performance which had a clear impact on service delivery.

Thus, despite sharing a common culture, history, and level of economic development, the contemporary approach to service delivery is strikingly different between the two states. The differences in political and administrative strategies across the two neighbouring states, which until their bifurcation were part of the same administrative structure, provide an almost unique set of conditions to examine the impact of different institutional contexts on voter responsiveness to clientelism. Importantly, we can be confident that the variation in the delivery of public services across the states is not endogenous to local electoral or socioeconomic factors but rather is a consequence of the different reform strategies adopted by political leaders. In other words, it is not an underlying shift in voter responsiveness to clientelism that has pushed one state in a more programmatic direction in some areas of government activity, but rather a strategic decision by the political leadership in Chhattisgarh to make the public delivery of welfare programmes work more successfully.

## Data Description

In order to examine the impact of these different institutional contexts on citizen responsiveness to clientelist appeals, we administered a survey in 40 villages on either side of the new state border: 20 in Madhya Pradesh (in the assembly constituencies of Anuppur and Kotma) and 20 in Chhattisgarh (in the assembly constituencies of Marwahi and Manendragarh). Their location is shown in Fig. 1. We purposively selected the two state legislative assembly constituencies on either side of the border matching the incumbency status of each, so that we had both a BJP- and Congress-held constituency in each state. Within each constituency, we randomly selected 10 villages, and in each village, we randomly selected 12 people from the electoral rolls. The surveys were completed during the election season of November–December 2013 during which new state legislative assemblies were elected simultaneously in both states. The surveys were conducted after voting had taken place but before the results were announced so that participants would be thinking about the conduct of the recent elections but not influenced by their outcomes.

Table 2 presents a balance check on factors that are theoretically thought to be related to vote buying. By far and away, the two most important factors that have been identified in the literature are wealth and education. Poor people and poorly educated people are thought to be more willing to sell their vote. As the table shows, our two groups are well balanced, and selected inhabitants on either side of the border are statistically indistinguishable on these covariates. Moreover, what differences there are work against our key hypothesis as respondents on the Madhya Pradesh side of the border are slightly better educated and slightly better off. Other possible confounds are cultural norms (Auyero 2000) and ethnicity (Chandra 2004). Culturally, the two groups are very similar: the villages on either side of the border are in deep forested areas. Inhabitants are predominantly Hindus with large Scheduled Tribe populations. Despite these similarities, we should note that there are more Scheduled Tribes, specifically from the Gond community, on the Chhattisgarh side of the border. However, if anything, we might expect Scheduled Tribes to be more likely to respond to clientelist appeals as they are one of the most economically deprived ethnic groups in India, so this lack of balance works against our key hypothesis.

**Table 2:** Balance tests on covariates

	Chhattisgarh	MP	*T* test (diff. of means	*P* value
Median HH income per month	Rs3000	Rs3000		
Mean HH income per month	Rs9141	Rs11551	1.55	0.121
Literacy rate	62	68	1.566	0.118
Female	49	48	0.136	0.892
Mean age	37	40	2.586	0.010
Hindu	86	94	2.90	0.003
ST	48	22	6.21	< 0.0005
Gond	37	16	5.33	< 0.0005
Village	43	43	0.132	0.895
N	239	240		

Lastly, institutional factors such as regime type, electoral systems, or ballot design (Golden 2003) and political competition (Kitschelt and Wilkinson 2007) are often thought to be related to clientelism. Institutional factors are obviously the same for both groups since all Indian states follow the same electoral systems. In addition, the structure of political competition is also the same: the BJP are the incumbent state government in both states; the principal party competition is between the Congress and BJP in each of the selected constituencies; and there was an incumbent MLA (Member of the Legislative Assembly) from both the Congress and BJP on each side of the border.

Given that a natural experiment is an extremely difficult (if not impossible) research design to execute for our research question, our data on the history of state formation, public service reform, and balance tests on theoretically relevant covariates give us confidence that our two sample groups do provide valid counterfactual groups. That said, in order to improve our causal inferences, we first employ regression analysis to control for any differences that do exist between our two groups, and second, we turn to semiparametric matching methods to balance covariates to mitigate against possible confounders (Ho et al. 2007).

## Institutional Performance

To gather data on the institutional context, we asked a range of questions designed to measure how people access and evaluate a range of different public services on the ground. As already mentioned, the areas of public service delivery that have undergone the most extensive process of reform in Chhattisgarh are concerned with food and employment. If these institutional reforms have been implemented effectively on the ground, then we should anticipate that evaluations of these public services will markedly differ between the two states. By contrast, those services which have not been reformed—and so share the same institutional legacy—should function—and be evaluated—in a similar way.

From Table 3, we can see that by far and away the biggest differences between the states are on evaluations of food (PDS) and employment (MGNREGS). In both cases, the services are evaluated far more positively in Chhattisgarh than they are in Madhya Pradesh. This indicates that the reform process has shaped the ways in which people experience and evaluate the services on the ground. By contrast, those services which have not been subject to major reform are evaluated in a very similar way across the two states. There are not any significant differences between our two groups on evaluations of health, education, electricity, water, or law and order. The only exception is for roads, where evaluations do differ significantly between the two states, although the magnitude of the difference (0.23 points) is substantially less than the mean difference for evaluations of the PDS, which is nearly three times greater at 0.63 points.

**Table 3:** Evaluations of public services

	Chhattisgarh	MP	Difference of means (*T* test)	*p* value
Roads	1.54	1.77	3.00	0.002
Health	1.81	1.72	1.22	0.223
Electricity	1.54	1.50	0.04	0.621
Water	1.99	1.92	0.86	0.389
Law and order	1.79	1.77	0.31	0.755
Education	1.39	1.52	1.91	0.056
Food (PDS)	1.14	1.77	9.49	< 0.0005
Employment (MGNREGS)	1.76	2.15	4.87	< 0.0005
*N*	231	214		

Now, thinking about how things have changed over the last 5 years. Please tell me whether you think each of the following have got better, stayed the same, or got worse? (where 1 = got better; 2 = stayed the same; 3 = got worse)

To get a deeper understanding about why these performance evaluations differ between the two groups, we asked a series of follow-up questions specifically about the PDS and MGNREG schemes (Table 4). The first thing to notice is that despite similar levels of poverty between the two groups, access to ration cards—and crucially access to the BPL ration cards—is much lower on the MP side of the border. Whereas 79% of inhabitants on the Chhattisgarh side had a BPL card, just 34% on the MP side did so. Moreover, those people who did possess a BPL card in MP were not disproportionately found among the most needy, and in fact possession of a BPL card was somewhat higher among the better off than it was among the extreme poor. In follow-up interviews, we asked inhabitants why they did not have a ration card (if they did not have one) and we were frequently told that it was because they had not paid a bribe to the local bureaucrat. One popular refrain we heard in the villages in MP was that ‘here the rich people have ration cards but the poor people don’t’. Corruption in the local bureaucracy was seen to be rife and a major obstacle to the successful implementation of policies. Others told us that although the Chief Minister in MP had lots of good initiatives, these policies never worked well on the ground. This is borne out by our survey evidence which shows that access to the PDS is much higher on the Chhattisgarh side of the border than on the MP side, as is satisfaction with how well the scheme works.

**Table 4:** Access to public services and evaluations

	Chhattisgarh	Madhya Pradesh	*T* test	*p*
Possession of ration card	94	81	4.52	< 0.0005
Possession of BPL card	79	34	11.10	< 0.0005
Recipient of PDS	92	46	12.58	< 0.0005
Recipient of MGNREGA	44	28	3.50	< 0.0005
Satisfaction with PDS	1.54	3.03	12.48	< 0.0005

Final row, figures show responses to question: ‘All in all, how satisfied or dissatisfied would you say you are with the way in which the PDS runs nowadays?’ (1 = very satisfied; 2 = quite satisfied; 3 = neither satisfied nor dissatisfied; 4 = quite dissatisfied; 5 = very dissatisfied)

These results are reassuring for the validity of our comparison. We can be confident that the different institutional environments are a consequence of specific reforms to service delivery that were carried out by the political leadership in Chhattisgarh on the PDS (and to a lesser extent MGNREGS), rather than more general differences between administrative zones and local bureaucracies which may be related to historical legacies from before the bifurcation of the state.

## Institutional Performance and Responsiveness to Clientelism

Having established the broad equivalence of our two groups on all theoretically important confounds, and described the process of top-down reform which has led to very different institutional environments, we now turn to examining the impact of the institutional context on voters’ willingness to vote in return for particularistic material goods. To this end, we asked respondents in our survey about a number of hypothetical vote buying situations. We carried out a split sample survey experiment where the party was randomised so that half of the sample was asked about a BJP party worker and half the sample was asked about a Congress party worker. We piloted various different versions of the questionnaire to see how respondents reacted to different phrasings of the question. Given that clientelism is such a pervasive feature of Indian politics, and that vote buying was discussed quite openly by inhabitants of the villages, we decided to ask a simple and direct question that was easily understood. We asked four variations of the question, in which the value of the hypothetical inducement varied from very small (vegetables) to very large (a government job).

Now, I’d like you to imagine that during the recent election campaign, a party worker from the Congress/BJP gave you money that would allow you to buy vegetables for your family for a week. Would you vote for their party?

And what if someone from Congress/BJP helped to pay the expenses for medical treatment for someone in your family? Would you vote for their party?

And what if someone from Congress/BJP helped get your house a new water pump? Would you vote for their party?

And what if someone from Congress/BJP helped to get a member of your family a government job after the election? Would you vote for their party?

The first thing to note is that respondents had a clear ordering of the value of the inducements. People were most responsive to large inducements and least responsive to small inducements. As we would expect, the provision of a job was the most powerful inducement to vote for a party, with nearly 60% of respondents reporting that they would vote for the party providing the material favour. The provision of a water pump was valued slightly more highly (26%) than medicine (18%). The provision of vegetables was valued somewhat less, but even in return for a relatively minor inducement, around one in ten people still said that they would vote for the party who provided the material favour. These findings show that people living in the context of rural poverty, unemployment, and low rates of literacy are highly responsive to clientelist appeals.

In order to test the impact of the institutional context on voters’ responsiveness to material inducements, we run a series of logistic regression models that also control for individual-level attributes. In particular, it is well known that poor and uneducated voters are most receptive to clientelist appeals, but does the institutional context also matter when we take into account these individual-level attributes?

Table 5 shows the parameter estimates for the most theoretically important covariates of vote selling. In each model, the dependent variable is whether someone would vote for a party in return for the named inducement on offer (1 = Yes; 0 = No). The independent variables are institutional context (where 1 = Chhattisgarh side of the border; 0 = MP side of the border); poverty (where 1 = above poverty line; 0 = below poverty line, and poverty line is set at a family living on less than £1 per day); education (where 1 = above primary education; 0 = primary or below); caste (which captures the main ethnic groups living in the locality and distinguishes between Upper Castes, OBC (Other Backward Classes), SC (Scheduled Castes or former untouchables), ST (Scheduled Tribes or ‘indigenous’ population) and others); and co-partisanship. Our split sample survey experiment randomised the party offering the inducement. In a separate question, we asked respondents about their own party affiliation. The variable for co-partisanship links these two questions together (where 1 = party offering the inducement matches the voter’s party affiliation and 0 = it does not match).

**Table 5:** Responsiveness to inducements, logistic regression

	Model 1: vegetables		Model 2: medical		Model 3: water pump		Model 4: job	
	B	SE	B	SE	B	SE	B	SE
Institutional context	1.232***	0.410	− 0.009	0.269	0.041	0.239	− 0.090	0.211
Poverty (below = Ref)								
Above poverty line	− 0.955*	0.496	− 0.506	0.310	− 0.372	0.268	− 0.102	0.224
DK	0.064	0.485	0.127	0.330	0.404	0.297	− 0.093	0.298
Education	− 0.900*	0.477	− 0.672**	0.301	− 0.435*	0.257	− 0.504**	0.220
Caste (upper = Ref)								
OBC	1.052	0.811	0.630	0.502	0.659	0.407	0.701**	0.289
SC	1.058	0.911	0.825	0.578	0.626	0.494	0.497	0.376
ST	1.612**	0.806	1.147**	0.499	1.266***	0.411	1.120***	0.316
Other	1.394	1.067	1.112*	0.627	0.812	0.548	− 0.118	0.428
Co-partisanship	0.690*	0.368	0.678***	0.258	0.458**	0.232	0.406*	0.212
Constant	− 1.231***	0.410	− 2.174***	0.691	− 1.864***	0.232	− 0.013	0.485
LR chi^2^ (9)	33.10***		35.07***		39.01		41.93***	

*N* = 479

*p < 0.10; **p < 0.05; ***p < 0.01

In line with prior theory, we can see that poverty and education influence whether or not people respond to clientelist appeals. Across all models, the variables are correctly signed. People living above the poverty line and people with some education above primary are less responsive to clientelist appeals (particularly in the case when the size of the inducement is very small). In line with prior theory, we also observe evidence consistent with the diminishing marginal utility of such inducements. The magnitude of the coefficients for poverty and education is smaller for large inducements (and do not achieve significance) than they are for small inducements (which are significant), indicating that the greater the value of the good voters are offered, the less difference there is between whether richer people and poorer people are responsive to the inducement. We can also see that there are some ethnic differences—and Scheduled Tribes—one of the most deprived groups in India—are more responsive to clientelist appeals than the other castes. This finding is consistent across the models.

Interestingly, we also see strong effects with respect to partisanship. Across each of the models, voters are more responsive to inducements that come from co-partisans, though once again the magnitude of the coefficient is greater for small inducements than it is for larger inducements. When the effect of co-partisanship is large, then the inducement to vote has a stronger effect on co-partisans than it does on non-supporters, meaning that the inducement has a relatively stronger impact on mobilisation than conversion. By contrast, when the effect of co-partisanship is small, then the inducement is able to both mobilise and convert equally. This implies that small inducements are more effective at mobilisation than conversion, and that parties may get more bang for their buck by targeting supporters with small inducements and ‘buying turnout’ (Nichter 2008) rather than offering these inducements to swing voters or partisans of other parties.

However, even controlling for all these factors, there are significant differences between the two institutional groups in terms of how responsive people are to the different types of inducement. In the context of poor institutional performance on the MP side of the border, respondents are significantly more responsive to small inducements than they are in the well-performing institutional context on the Chhattisgarh side. However, as the size of the inducement increases, the difference between the two groups decreases to non-significant levels. When the inducement is a water pump or medicine, we do not see any significant institutional effects. Both of these goods are quite highly valued goods. Most of the villages on both sides of the border only had a limited water supply, and during fieldwork people frequently raised healthcare and medical expenses as a major source of anxiety (see also Krishna 2011). Consistent with our theory then, we observe relatively larger institutional effects on small inducements than on medium and large inducements, which suggests that the institutional context can influence the marginal utility of vote buying.

We can get a clearer idea of the magnitude of these effects by calculating the predicted probabilities for whether a person from the Scheduled Tribe community, living below the poverty line with no educational qualifications, would vote for a party in return for the named inducement on offer, according to which side of the border they live (Table 6). For this group of voters, the predicted probability of responding to the food inducement is 0.26 in the context of poor institutional performance on the MP side of the border but just 0.09 in the well-performing institutional context on the Chhattisgarh side. This represents a sizeable difference. Moreover, the average effect of institutional context (when holding all other variables at their mean) is about 9 percentage points.

## Propensity Score Matching

In order to strengthen our inferences, we pre-process the data with a ‘matching’ procedure (e.g. Dunning 2008; Ho et al. 2007). Under this procedure, the effect of being exposed to different institutional contexts is more accurately measured by comparing the attitudes and behaviours of survey respondents who are similar to one another, save the fact that one was exposed to a better-performing public service and the other was not. In other words, the idea is that the researcher imposes some degree of ‘experimental’ control on what is observational data (Klofstad et al. 2013). By comparing the attitudes and behaviours of similar individuals who were and were not exposed to a well-performing public services, we can be more confident that any observed differences in attitudes and behaviours between these groups are unrelated to the factors that the respondents were matched on (and, as such, are a consequence of being exposed to a well-performing public services instead of some confounding factor).

In seminal work, Rosenbaum and Rubin (1983) proposed propensity score matching as a method to reduce the bias in the estimation of ‘treatment’ effects with observational data sets. Matching methods differ in the way matched cases between the study groups are defined (Ho et al. 2007). Inverse probability weighting with regression adjustment (IPWRA) estimators model both the outcome and the selection, which means that the estimate of the institutional effect will be unbiased if either the selection model or the outcome model is mis-specified. It is important to note that matching is less precise than a controlled experiment because the procedure does not account for *unobserved differences* between individuals who were and were not exposed to different institutional contexts (Sekhon 2009). However, since unobserved differences between individuals who were and were not exposed to different institutional contexts are likely to correlate with observed differences, they are accounted for by proxy in the matching procedure (Stuart and Green 2008). To this end, an extensive set of covariates were used in the matching procedure, increasing the likelihood that any meaningful covariates of responsiveness to clientelism are accounted for in the analysis. Each of the covariates reported in Table 5 were included in the matching procedure. Importantly, we also included whether respondents had access to the PDS scheme (reported in Table 4) since we wanted to get an estimate of the institutional context, regardless of whether households across the two units had access to food benefits.

**Table 6:** Responsiveness to inducements: predicted probabilities

	Chhattisgarh	Madhya Pradesh
Vegetables	9	26
Medicine	29	30
Water pump	38	40
Job	75	74
*N*	239	240

Predicted probabilities for an ST living below the poverty line, with no education

To summarise the causal effect of institutional context, we can estimate the average treatment effect (ATE). Table 7 shows the ATE of institutional context on each of the inducements. Once again, we can see that the main findings hold up. The effect of being exposed to the better-performing institutional context of Chhattisgarh significantly reduces voters’ responsiveness to low-value inducements. The ATE is just under 6 percentage points. This is somewhat lower than the naïve estimate from the logistic regression models, of about 9 percentage points. However, once again, we do not find evidence that the institutional context influences voters’ responsiveness to higher-value inducements which may be standing in for public goods and services that are in scarcer supply in both states.

**Table 7:** Average treatment effect, inverse-probability-weighted regression-adjustment

	Coeff.	Robust std. err.
Vegetables	− 0.055**	0.022
Medicine	0.041	0.038
Water pump	0.045	0.041
Job	0.017	0.057

*N* = 479

*p < 0.10; **p < 0.05; ***p < 0.01

## Conclusion

When do voters ‘sell their vote’? Answers to this simple question have long puzzled scholars of comparative politics. Although a wide range of individual-level factors to do with income and education have been proposed, up until now there has been relatively little attention on how the calculus of voters is influenced by the institutional context and the delivery of public services. Part of the reason for this is that the vast majority of studies on clientelism have been based on single country case studies. Comparative studies have been few, and those which do exist have tended to focus on the behaviour of clientelist parties rather than the responsiveness of voters.

In this study, we have attempted to overcome these difficulties by drawing on a carefully constructed comparison made possible by the division of Madhya Pradesh into two separate states which have pursued very different processes of public service reform. This allows us to examine two very different institutional contexts which share many social, economic, and political characteristics. To our knowledge, this is the first study that has managed to examine how both individual-level factors and institutional factors jointly shape whether or not people respond to clientelist appeals.

At the individual level, our results are supportive of current theories of vote buying which emphasise the importance of poverty and education. In addition, we show that mobilisation effects are greater than conversion effects, and that small inducements have a greater impact on mobilisation that conversion. However, we also show that above and beyond these individual-level factors, the institutional context matters. We show that institutional context can influence the marginal utility of vote buying. When public services function badly, people are prepared to sell their vote for relatively little, but as services perform better, the cost of vote buying also increases. Or put another way, the greater the value of the good voters are offered, the less difference we observe between whether people are prepared to sell their vote in well-performing institutional settings and badly performing institutional settings. This suggests a threshold effect. When institutions do not function well, even small inducements can have a sizeable effect on vote choice. However, in better-performing institutional contexts where basic services are better provided, such minor inducements are less likely to be successful at buying votes. Thus, as institutions perform better, the cost of vote buying also increases.

The central implications of these findings are that citizens respond to clientelist appeals because they are risk averse, relying on short causal chains that prize direct, instant clientelist benefits over indirect, programmatic linkages promising uncertain and distant rewards to voters. Although it is well known that poverty and education matter in this regard, our findings show that institutional context also matters. If institutions do not function well, and are leaky, then the probability of ever receiving the promised benefit of a programmatic policy is extremely low. In this situation, rational voters will discount the future and the appeal of short term clientelist goods will be more attractive. However, when institutions function well, even in a limited way, voters can see the link between policy promises and policy implementation and so will be less likely to sacrifice their preferred policy outcome for a short-term pay-off. Poor institutional performance therefore makes the prospect of direct personal transfers today more attractive than the promise of redistributive public policy tomorrow.

## Footnotes

1 The bureaucratic agencies that are involved in the performance of a programme such as the Public Distribution System operate at multiple levels: the local, state, and more distant federal levels. At the state level, they may include the functioning of the Department of Food and Civil Supplies, Cooperatives Department, State Civil Supplies Corporation, the Food Corporation of India, Warehousing Corporation of India, and National Informatics Centre. The distribution of Below Poverty Line and ‘ration’ cards, which determine eligibility for this programme and others, depends on the office of the District collector and Panchayat secretary at village level, as well as oversight by elected members of local bodies.

2 Census of India 2001. Because the assembly constituencies surveyed cross more than one sub-district, it is not possible to provide census data at the level of the assembly constituency. The data here thus refers to districts that are larger than the assembly constituencies surveyed. In the villages we surveyed within assembly constituencies, as shown below (Table 2), the Scheduled Tribe population was in fact larger on the Chhattisgarh side of the border.

3 Fieldwork for this study was conducted before the National Food Security Act came into effect in the states.

4 The reform process is analysed in greater depth in Tillin et al. (2015) drawing on interviews with politicians, officials, NGO staff, and other local informants in both states.
